# Exploring the Effectiveness of Immersive Video for Training Decision-Making Capability in Elite, Youth Basketball Players

**DOI:** 10.3389/fpsyg.2018.02315

**Published:** 2018-11-27

**Authors:** Derek Panchuk, Markus J. Klusemann, Stephen M. Hadlow

**Affiliations:** ^1^Movement Science, Australian Institute of Sport, Canberra, ACT, Australia; ^2^Basketball Australia Centre of Excellence, Canberra, ACT, Australia; ^3^School of Human Movement and Nutrition Sciences, The University of Queensland, Brisbane, QLD, Australia

**Keywords:** skill acquisition, expertise, decision-making, perceptual-cognitive training, immersive video, team sports, basketball

## Abstract

Decision-making is an essential capability for success in team sport athletes. Good decision-making is underpinned by perceptual-cognitive skills that allow athletes to assess the environment and choose the correct choice from a number of alternatives. Previous research has demonstrated that decision-making can be trained “off-line” by exposing athletes to gameplay scenarios and having them make decisions based on the information presented to them. These scenarios are typically presented on television monitors or using life-size projections but recent advances in immersive video capabilities provide opportunities to improve the fidelity of training by presenting a realistic, 360° view of the competition environment. The purpose of this study was to assess the effectiveness of immersive video training and whether training would improve decision-making performance in elite, youth basketball players (male and female). A training group completed 10 or 12 immersive video (360° video presented in a head-mounted display) training sessions in which they viewed and responded to gameplay scenarios across 3-weeks while the control group only participated in their usual training routine. Performance was assessed on an immersive video test and during small-sided games (SSG). The male training group had a large, non-significant improvement on immersive test score (+4.0 points) and in the SSG (+5.8 points) compared to the male control group (+0.3 points and +1.0 points, respectively). While both the female control group (+9.7 points) and training group (7.4 points) had large improvements in the immersive training test, only the female control improved their performance in the SSG (+6.9 points). Despite the mixed findings, there may be benefit for using immersive video for training decision-making skill in team sports. The implications of these findings (e.g., gender of the actors used to create stimuli, variety of scenarios presented) and the limitations of the experiment are discussed.

## Introduction

Good decision-makers are highly sought after in team sports yet a precise characterization of what makes a good decision-maker in a particular sport is rather elusive. Decision-making is defined as the process of choosing one option from a group of alternatives (Bar-Eli et al., 2011) and effective decision-making can be the difference between success and failure in team sports. In the expertise literature it is well established that elite decision-makers, while often indistinguishable from other performers on physical attributes, possess superior perceptual cognitive skills compared to their near-elite and novice counterparts (Mann et al., 2007). Elite decision-makers have better pattern recognition and recall skills (Gorman et al., 2012), anticipation (Müller and Abernethy, 2012), different visual search strategies (Klostermann et al., 2018), and knowledge structures (Sutton and McIlwain, 2015) which underpin their superior decision-making capabilities. Given that these perceptual cognitive skills discriminate between elite, near-elite, and novice performers it could be assumed that these skills can be trained and this training would then transfer into improved on-field performance (Abernethy and Wood, 2001). The purpose of this experiment was to explore whether new technology that allows for the capture of immersive video could be used to train decision-making in elite, youth basketball players.

Decision-making in sport has typically been assessed and trained using simulations of sport-related scenarios presented to participants using television/computer monitors (e.g., Lorains et al., 2013) or through projection of life-size images (e.g., Bruce et al., 2012). While the size of the image has no influence on the decision making performance of athletes (Spittle et al., 2010), these studies have consistently shown differences in decision-making skill between experts and novices in sports such as: netball (Bruce et al., 2012), baseball (Paull and Glencross, 1997), soccer (Vaeyens et al., 2007), and basketball (Ryu et al., 2013). More importantly, there is growing evidence that perceptual-cognitive training can be used to improve the performance of athletes in competition (Williams et al., 2003; Gabbett et al., 2007). In basketball, the effects of perceptual-cognitive training for improving decision-making have been equivocal. Starkes and Lindley (1994) showed that perceptual-cognitive training could be used to improve response time and accuracy in youth, elite basketball players. While they didn’t find any transfer of training to on-court performance, it could be argued that the transfer test used – having players view live game scenarios from the stands – did not faithfully recreate the demands of a basketball game. More recently, Gorman and Farrow (2009) found no benefits for perceptual-cognitive training or transfer of training in skilled basketball players although there was a trend for players who underwent training to improve their performance on the video-based test.

Despite the lack of evidence to support the efficacy of perceptual-cognitive training in basketball, this mode of training can be an effective means of improving athlete performance across a range of skills (Larkin et al., 2015). According to the Modified Perceptual Training Framework (MPTF; Hadlow et al., 2018), the efficacy of any perceptual training tool can be assessed by examining the targeted perceptual function (e.g., basic ocular function vs. decision-making), how closely the stimuli resembles and behaves like stimuli from the competition environment, and whether the response required mimics the demands placed on performers in the competition. The emergence of technology that improves the fidelity of the simulations being presented to athletes offers promising opportunities for the development of future training approaches (Craig, 2013). For example, advances in virtual reality (VR) have already demonstrated the added benefit of having athletes perform in an interactive, virtual environment compared to video images (Vignais et al., 2015). Because this type of training targets high-order processes, presents sport-specific stimuli, and requires sport-specific responses, the MPTF would predict benefits from training would transfer to on-field performance. A recent study by Gray (2017) highlighted the benefit of VR training in baseball; players who underwent an adaptive virtual training program improved their performance on a batting test and in competition.

While VR training is certainly a promising avenue for improving sports performance, it is currently not practical for many sports teams. Hardware to support VR training (e.g., Oculus Rift, HTC Vive) is more affordable but the specialist software to support training programs (i.e., sport-specific scenarios that the performer interacts with) requires resources (i.e., programming and development) that may be beyond the means of many organizations. A possible solution could be the use of immersive video that maintains some of the benefits of VR but is not as resource intensive. Commercially available 360° video cameras and head-mounted displays (e.g., Google Cardboard, Samsung Gear VR) now permit the relatively easy creation of immersive video content. For example, a 360° camera could be used to capture sport-specific scenarios from a first-person perspective and these could be played back on the mobile phone of the athlete. The MPTF would predict that this type of training would produce better transfer than viewing scenarios on a monitor/projector screen because of the increased response correspondence. Rather than viewing from a static point-of-view, the performer now has the ability to control the viewing orientation. Given that the head is an important component of the gaze control system (along with the eyes and body; Vickers, 2007) this additional level of interaction may improve performance outcomes over traditional training approaches which only permit a single perspective.

Opportunities to create more realistic and interactive perceptual-cognitive training environments are becoming increasingly accessible with the development of technology. While previous research using video monitors has been shown to be effective for improving performance, little is known about whether emerging technology is as effective. The purpose of this study was to explore whether immersive video could be used to improve the decision-making performance of elite, youth basketball players and whether training using immersive video would transfer to improved passing performance in small-sided games. We hypothesized that players who underwent immersive video training would improve their test scores and performance in small-sided games relative to a control who participated in regular training only.

## Materials and Methods

### Participants

Twenty (*n* = 20; 10 male, 10 female, age: 17.0 ± 0.6 years) elite, youth basketball players (positions: 6 guards, 6 wings, 8 bigs) volunteered to participate in the experiment. All players were members of the National Under-19 Basketball Australia Centre of Excellence basketball program at the time of testing and had represented their country at an international competition. One participant was unable to participate in the experiment due to an injury and another was unable to complete any of the testing due to motion sickness induced by wearing the head-mounted display (the participant indicated a history of hyper-susceptibility to motion sickness). This left the final number of participants at 18 (9 female, 9 male). Due to coaches requests for players to complete the training the final group composition was 11 training (5 males, 6 females) and 7 control (3 female, 4 male). This study was carried out with the recommendations of the National Health and Medical Research Council’s Statement on Human Experimentation and Supplementary Notes, NHMRC Australian Health Ethics Committee. The protocol was approved by the Australian Institute of Sport Ethics Committee. All participants or their guardians gave written informed consent in accordance with the Declaration of Helsinki.

### Apparatus

#### Immersive Video Capture

Immersive videos for testing and training were created by filming basketball game play scenarios using a 360° video recording system on the court. The recording system consisted of six action cameras (GoPro Hero 4 Black, GoPro, Inc.) mounted on a camera rig (Freedom 360 Classic Mount; Freedom360, LLC) that had each camera facing a different direction. The camera rig was attached to a microphone boom stand (Manfrotto 420B Combi Boom Stand, Manfrotto) and mounted on a dolly (Manfrotto 127 Portable Dolly, Manfrotto). This allowed for the camera to be held just over the player’s heads while the scenarios were being filmed and to move with them. The perspective captured in the videos was from a first-person viewpoint; if the participant looked down in the video they could see the player in control of the ball. During testing and training, however, looking down would prevent the participant from viewing the unfolding scenario in front of them and none of the participants adopted this strategy during testing. Prior to filming all of the cameras were set to record and an audio cue (a clap captured on all six cameras) was used for later synchronization.

#### Scenario Filming

Filming was done during a 3-h session on a regulation basketball court (with shot clock) in a stadium setting. Ten players from the men’s basketball team (two were participants in the study, however, filming occurred 3 months prior to testing and performance on the test was at or below the group average which suggests that there was a significant wash-out period between filming and testing) were used as actors in the filming session. They were dressed in their game uniforms and half the players wore the home uniform (yellow) and the other half wore the away uniform (green). Prior to filming players were informed about the goals of the session and given the opportunity to practice how each scenario would proceed. The scenarios were created by one of the co-authors (MK; who was an assistant coach with the men’s program) and agreed upon by two other members of the coaching staff). A total 56 scenarios were created for filming and these included variations of 6 different base formations that were used by the team. Each scenario was designed to include ball movement prior to a designated decision-maker receiving the ball and a clear option for a pass (i.e., there was an open teammate). We included a pre-defined option to ensure that players involved in the filming had a clear goal. To avoid making scenarios too position-specific, a wide variety of scenarios for post players and guards, as well as generic passing decisions that could occur across multiple positions were included. Prior to filming each scenario both teams were told what the desired outcome was and how they were to respond to the ball movement. They were then given the opportunity to simulate the play once before filming commenced. For filming the players were told to play at full speed as though they were in a competitive game. A camera operator moved the camera based on the designated decision-maker’s movements and a “spotter” viewed the entire sequence to ensure that the camera was over the players head while filming. If there were errors in ball movement or the camera operator could not keep the camera over the designated decision-maker’s head during filming, the scenario was repeated. At least two good takes (as noted by the spotter) were captured for each scenario.

#### Immersive Video Creation

**FIGURE 1:**
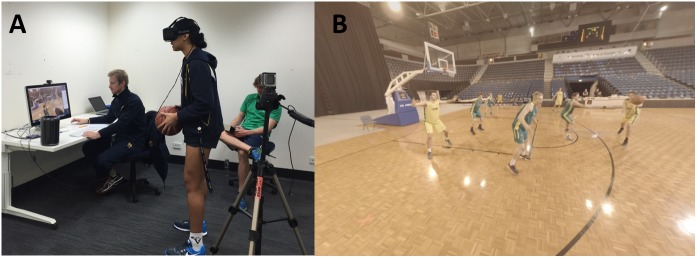
Example of the testing apparatus **(A)** and the players view in the HMD **(B)**.

Video footage from the six action cameras was synchronized in software (PluralEyes 4.1, Red Giant, LLC) and exported into a video stitching program (Autopano Video, Kolor). The software stitched the videos together and created a single 360° video that could be played back in a head-mounted display. Once the videos were created they were inspected for quality to ensure that the camera remained over the designated decision maker’s head to create a first-person perspective and that the correct decision was made. One of the coaches also viewed the clips to ensure they accurately captured the required movements for a given scenario. A total of fifty-six unique immersive video clips were created for inclusion into the study for testing and training (see Figure 1B for an example).

#### Testing Apparatus

A selection of fifteen clips was selected to be used exclusively in testing sessions (i.e., they were not presented during the training intervention). A minimum of two clips from each of the six broad categories of scenarios was included. For the testing sessions, video clips were presented through a tethered head-mounted display (HMD; Oculus Rift SDK, Oculus). This allowed the researchers to monitor through an attached display and record the responses of the participants (see Figure 1A). The HMD was connected to a computer (Mac Pro, Apple, Inc.) running the immersive video clips through a 360° video player (Kolor Eyes, Kolor). While wearing the HMD, the orientation of the video presented would change in response to head rotations of the player but did not respond to the movements (i.e., translations) of the player’s body otherwise (i.e., moving the head would cause the scene to rotate but any other actions had no effect). A video camera (GoPro Hero 4 Black, GoPro) was positioned to capture the actions and audio from the player wearing the headset and the orientation of the video presented on the computer screen.

#### Training Apparatus

Immersive video clips not used in the testing session (41 clips) were used for the training intervention. For the training sessions players viewed 360° video footage via a mobile HMD (Samsung Gear VR, Samsung) presented on a mobile phone (Samsung S6). To ensure consistency between the testing display and the training display, the videos were presented at the same resolution. While this prevented data collection during training (i.e., during testing sessions the only difference was that the researchers could view the player’s head orientation) it allowed sessions to be conducted at the basketball court prior to normal practices. The footage was presented using a custom designed video player. The video player used a text script that contained the name of the clip, the start time of the clip, the decision time (i.e., when the decision-maker passed the ball), and the end time. Using this information, the video player would present the video at the designated start time. The participant could then orient themselves to the information available within the scenario and then press a button on the side of the HMD to start the video. The video would play until the point of the decision when the video would pause and the participant would be asked to make a decision. Then they would press the button on the side of HMD again at which point the next scenario would be presented (note: in the training sessions, the videos ended at the point of decision and no additional information or feedback was provided).

#### Small-Sided Games (SSG)

To determine whether transfer of training occurred, SSG were used to assess player’s on-court performance. For the SSG, players were split into two teams of four players each (4 vs. 4) and competed on half a basketball court. The structure of the games was two 5-min halves and otherwise played according to the official FIBA 3 vs. 3 game rules^1^. SSG were video recorded and the footage was analyzed using SportsCode Elite (Hudl). On every occasion that a player had possession their performance was assessed against the categories shown in Table 1. Each category was weighted according to its value toward a positive outcome (e.g., scoring a basket) or negative outcome (e.g., contested shot). Player performance was coded and the points from each category added together to give a total score after each SSG for each player. This method was used by the team to assess player performance during games and allowed for easier comparisons to other performances.

**Table 1:** Decision-making categories for assessing performance during the SSG.

Category	Description	Points
Successful pass	A pass that arrives at the intended teammate	1
Hockey assist	A pass that leads to an assist (e.g., the next pass results in an assist) or causes defensive perturbations. A pass from the inside out (kickout passes), an extra pass to an open player or a pass into an inside player are examples of hockey assists.	2
Assist	A pass that directly leads to the team mate scoring	3
Open shot	The decision to recognize the opponent is more than 2 m away and one is in a position to score	3
Contested shot	The decision to shoot despite an opponent being close	-1
Deflected pass/bobble	A pass that is deflected or is not delivered accurately to a team mate	-1
Passing turnover	A pass that is stolen by the opposition or thrown out of bounds	-2
Dribble turnover	When the opponent gains possession while the attacker is trying to dribble the ball	-2

### Procedures

After providing informed consent in the week prior to undergoing the testing and training intervention, participant’s on-court performance was assessed in two SSG conducted 48–72 h apart. In the following week, all participants completed an immersive pre-test session in the laboratory. The laboratory was an open space with a computer desk and video camera that permitted movement within the length of the tether to the HMD (4 m). For testing, the procedures and task were explained to the participant and then they were fitted with the HMD. The instructions for the participant were to: imagine they were in the shoes of the player in the clip, view the scenario as it played out on the footage, and make any decision (e.g., shoot, pass, dribble, etc.) that they liked when the ball came to them. If the clip stopped, then they were to make a decision as quickly as possible. After the instructions, they were then presented with five practice clips (selected from the training footage) to familiarize themselves with the procedure. Each scenario started with the clip paused and the participant was instructed to look around to orient themselves to the location of the other players and the ball. When they felt they were ready, they were asked to say “go” and one of the researchers started the video. Participants were asked to verbalize their decision as soon as they could and were given a ball to simulate their decision (e.g., if they decided to pass they would act as if they were intending to pass the ball in a particular direction – although the ball was not actually passed – if they decided to shoot they would mimic a shooting action). Once the practice clips were completed, participants were asked if they had any questions regarding the procedure and the instructions were provided again for reinforcement prior to presentation of the test videos. The test itself consisted of 15 unique clips presented in a randomized order for each participant. During the test participants were prompted with instructions if they were indecisive or failed to act out their decision. The test took 15 min to complete.

The training intervention started the week after the initial testing session. For the training intervention, participants were assigned to either the control group or training group. Both groups took part in their normal practice routine but the control group only participated in the SSG and testing sessions. In addition to this, the intervention group viewed 15 randomly selected immersive videos prior to their regular training through a HMD. Training was conducted on-court and the task and instructions were identical to that of the testing session. Each training session took 5 min to complete and was supervised by one of the researchers to ensure compliance with instructions. Due to scheduling constraints the female participants completed 10 training sessions over 3 weeks while the male participants completed 12 training sessions (number of sessions completed was used as a covariate in the analysis). The week after training completed, all participants completed an immersive test using the same videos as in the pre-test and competed in 1 or 2 SSG (due to injury issues and competition schedules the female participants only completed a single SSG). At the completion of the study, participants in the training group were also given a short survey that allowed them to provide feedback on the immersive videos and the training intervention.

### Dependent Variables

#### Immersive Test Performance

Player decisions were scored based on criteria established in consultation with three coaches from the Basketball Australia Centre of Excellence program. Coaches viewed the clips as many times as they liked and ranked their top 3 decisions (coaches were told they could choose any basketball-related decision and they were not limited to making passing decisions); each decision was then given a score between 1 and 3 with 3 being their preferred decision (Lorains et al., 2014). Despite designing the clips so there was a free player in each scenario (in accordance with the definition of decision making presented in the introduction), the open player was not always judged to be the best option by the coaches (as was expected). To account for this inconsistency, we summed the ranking between coaches to weight decisions where there was higher levels of agreement. For example, if all three coaches chose the same decision as their first preference it would be worth 9-points in the test and if one coached ranked a decision first and the other two ranked it third it would be worth 5-points in the test. Using this weighted system of scoring, a wide number of potential decisions were identified and the maximum score any player could get on the immersive test was 109-points.

#### SSG Performance

A total score was determined by tallying individual player results from each of the categories shown in Table 1 (i.e., the cumulative total of all the player’s decisions were tallied to provide a total score that could be positive or negative). Individual categories were also compared prior to and after the intervention.

### Analysis

Immersive test results and SSG performance variables (total score, eight individual categories) were analyzed separately using linear mixed modeling. In the model, the group (training, control), test (pre, post), and gender (male, female) were used as fixed factors, test was a repeated measure, number of training sessions and participant were used as random factors. The fit of the model was adjusted by inclusion of random intercepts and slopes and changing the variance structure. Goodness of fit between models was compared using the Akaike Information Criterion. Due to the exploratory nature of the study we performed *post hoc* tests for significant and non-significant effects using a Bonferroni correction and Cohen’s d was used to determine effect sizes (0.2 = small, 0.5 = medium, 0.8 = large). The data for total score (*w* = 0.967, *p* = 0.351) and SSG (*w* = 0.988, *p* = 0.850) were normally distributed).

## Results

### Immersive Test

**FIGURE 2:**
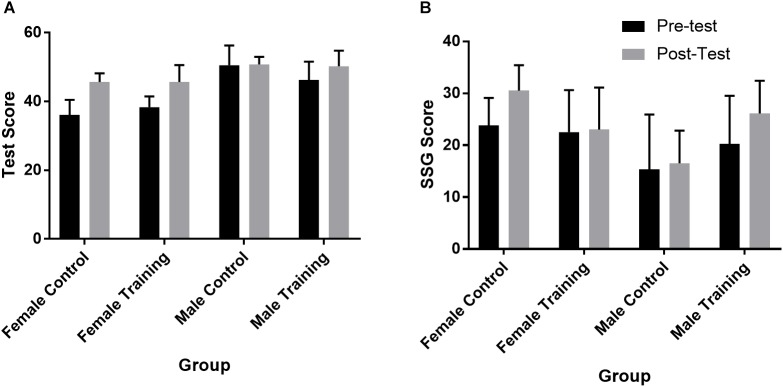
Comparison of pre-test and post-test scores for male and female control and training groups in the immersive video test **(A)** and in the small-sided games **(B)**. (Note: Errors bars represent SD).

The three-way interaction of group x test x gender was not significant (*p* = 0.275). Performance on the test is shown in Figure 2A and Table 2. For females, both the control (*p* = 0.007, *d* = 2.71) and training (*p* = 0.004, *d* = 1.79) groups had large, significant improvements. For males, the control group did not improve (*p* = 0.929, *d* = 0.06) while the training group had a large but non-significant improvement (*p* = 0.127, *d* = 0.80).

**Table 2:** Pre-test and post-test scores (mean ± SD) for the immersive test, total SSG score, and each individual variable from the SSG with a comparison of values (*p*-value) and effect size (d).

Variable	Gender	Group	Pre-test	Post-test	*P*-value	Effect size (d)
Immersive test score	Female	Control	36.0 ± 4.4	45.7 ± 2.5	0.007	2.71
		Training	38.3 ± 3.1	45.7 ± 4.9	0.004	1.79
	Male	Control	50.5 ± 5.8	50.8 ± 2.2	0.929	0.06
		Training	46.2 ± 5.4	50.2 ± 4.5	0.127	0.80
Total SSG score	Female	Control	23.8 ± 5.3	30.5 ± 4.9	0.262	1.31
		Training	22.5 ± 8.1	23.0 ± 8.1	0.855	0.06
	Male	Control	15.3 ± 10.6	16.5 ± 6.3	0.528	0.14
		Training	20.2 ± 9.3	26.1 ± 6.3	0.080	0.74
Successful pass	Female	Control	8.8 ± 4.1	6.0 ± 0.0	0.320	0.97
		Training	8.9 ± 4.6	9.3 ± 2.7	0.642	0.11
	Male	Control	4.7 ± 2.1	6.3 ± 3.3	0.173	0.58
		Training	4.1 ± 2.9	6.1 ± 5.9	0.218	0.43
Assist	Female	Control	1.8 ± 1.3	2.0 ± 1.4	0.858	0.15
		Training	1.8 ± 1.4	1.7 ± 1.6	0.862	0.07
	Male	Control	1.3 ± 1.4	2.1 ± 1.2	0.415	0.61
		Training	1.7 ± 1.6	1.5 ± 1.4	0.632	0.13
Hockey assist	Female	Control	1.7 ± 1.4	1.5 ± 0.7	0.726	0.18
		Training	1.7 ± 1.7	2.3 ± 2.0	0.216	0.32
	Male	Control	2.0 ± 2.2	0.9 ± 1.4	0.663	0.60
		Training	1.4 ± 1.7	1.8 ± 1.7	0.239	0.24
Open shot	Female	Control	3.7 ± 2.4	6.0 ± 0.0	0.104	1.36
		Training	3.1 ± 1.5	2.8 ± 1.0	0.777	0.24
	Male	Control	2.6 ± 1.7	2.1 ± 1.6	0.684	0.30
		Training	3.6 ± 2.6	5.8 ± 2.6	0.075	0.85
Contested shot	Female	Control	1.7 ± 2.1	0.5 ± 0.7	0.443	0.77
		Training	1.1 ± 1.1	2.2 ± 1.6	0.225	0.80
	Male	Control	2.7 ± 3.1	1.0 ± 0.8	0.050	0.75
		Training	1.1 ± 1.4	2.1 ± 1.7	0.340	0.64
Deflected pass/bobble	Female	Control	0.8 ± 1.2	0.0 ± 0.0	0.225	0.94
		Training	1.1 ± 0.9	0.7 ± 0.8	0.334	0.47
	Male	Control	0.3 ± 0.5	0.5 ± 0.8	0.575	0.30
		Training	0.7 ± 0.9	0.4 ± 0.7	0.493	0.37
Passing turnover	Female	Control	1.0 ± 0.6	1.0 ± 1.4	1.000	0.00
		Training	0.9 ± 1.2	0.8 ± 1.6	0.720	0.07
	Male	Control	0.7 ± 0.8	1.3 ± 1.3	0.286	0.56
		Training	0.3 ± 0.5	1.1 ± 0.8	0.130	1.20
Dribble turnover	Female	Control	0.2 ± 0.4	–	N/A	N/A
		Training	0.3 ± 0.5	–	N/A	N/A
	Male	Control	0.7 ± 0.6	0.1 ± 0.4	0.044	1.18
		Training	0.0 ± 0.0	0.3 ± 0.5	0.282	0.85

### Small-Sided Games

For total SSG score, the three-way interaction of group x test x gender was significant (*p* = 0.032). Performance in the SSG for each group and gender is shown in Figure 2B and Table 2. Follow-up tests did not reveal any significant differences. For females in the control group there was a large, non-significant improvement (*p* = 0.262, *d* = 1.31) in performance from the pre-test to the post-test while the female training group did not change (*p* = 0.855, *d* = 0.06). For males, there was no change in performance for the control group (*p* = 0.528, *d* = 0.14) while the training group had a medium-to-large, non-significant improvement in performance (*p* = 0.080, *d* = 0.74).

Individual variables from the SSG were compared and the results are shown in Table 2. For number of successful passes (*p* = 0.274), assists (*p* = 0.987), hockey assists (*p* = 0.910), contested shots (*p* = 0.713), deflected passes (*p* = 0.371), passing turnovers (*p* = 0.635), and dribbling turnovers (*p* = 0.056) there was no significant interaction of group x test x gender. For open shots, the three-way interaction was significant (*p* = 0.003). While *post hoc* tests did not reveal any significant differences, the female control group (*d* = 1.36) and male training group (*d* = 0.85) both had large increases in the number of open shots taken.

## Discussion

Sports teams are always looking for a competitive advantage and, in team sports, improved decision-making is viewed as an asset for athletes. In this study we sought to determine whether immersive video training could be effective for improving decision-making performance in elite, youth basketball players. Although we predicted that the training groups would show improvements in test and small-sided game performance, the results from our study were equivocal. When athletes were assessed on their decision-making performance, there were no differences in performance between the pre-test and post-test. Given the exploratory nature of the study and the fact that this was an applied study (i.e., coaches were interested in within-group changes), we analyzed the group differences and found that both the female control and training group and male training group had large improvements in decision-making performance on the immersive test (although the males improvement was non-significant). More importantly, we found a medium-to-large, non-significant improvement in overall performance during a SSG for the male training group and, rather unexpectedly, in the female control group – although there were limitations to the design which will be discussed later. Overall, there were some issues that may have affected the results but there was no detriments in performance as a result of using immersive video and, given the potential value of training observed, we would recommend using immersive video as a perceptual training tool although additional research is necessary to better understand how it compares to other training modalities.

One of the most striking findings is how differently males and females responded to the testing and training. While the male training group’s results were generally in line with expectations, the response of the female control was unexpected. Because the male control group did not show the same pattern of change as the female control group this could rule out test familiarity as a confounding factor. The simplest explanation may be that the training did not benefit females. There were, however, other design issues that may have influenced the results. First, there were only three participants in the female control group (vs. six in the training group) which limits the amount of data available for comparison. Second, the females only completed one SSG for the post-test which increases the likelihood that their performance during the SSG would be influenced by performance variables that may have temporally inflated their scores (Magill and Anderson, 2017). Third, the amount of training differed between groups; although the dose-response relationship is not well understood in perceptual-cognitive training (Larkin et al., 2015), it is possible, but not likely, that the two extra training sessions would have benefitted the male training group (although this doesn’t account for the improvements in the female control group). The content of the stimuli may have impacted the results as well. The footage used for testing and training was created using scenarios from the male’s team playbook and using male participants. Research into observational learning, based on social cognitive theory (Bandura, 1989), suggests that the similarity of a model to the participant (e.g., gender) can influence self-efficacy (George et al., 1992; Weeks et al., 2005) and motor performance (Meaney et al., 2005). Although this study did not assess observational learning, it is possible that using male actors in the stimuli may not have promoted the same level engagement and learning in female participants and future research may benefit from using actors of the same gender.

Feedback from the athletes was overwhelmingly positive. All of the athletes felt that the training was beneficial for improving their performance on-court with several commenting that the training “helped with court vision and being able to see options on offense” and “noticing where defenders were moving.” When asked about what they enjoyed about the training approximately half of the athletes commented on the realism of the scenarios, including comments such as: “it was really cool how real it felt” and “it felt like we were in the arena.” The added fidelity of the immersive videos (e.g., full environment, ability to control head orientation) may have been beneficial although it has been suggested that the realism of an immersive environment is less important when compared to whether or not an it maintains behavioral realism (Craig, 2013). In terms of aspects of the training that could be improved, approximately two-thirds felt that increasing the number/variety of scenarios and using scenarios with different visuals features (e.g., different environments, teams, etc.) would make the training more engaging. There may be skill learning advantages to increasing the variety of scenarios as well due to the contextual interference effect (Porter and Magill, 2010). The results from the immersive test were somewhat surprising given the variety of responses received from players (and coach raters) on the same scenarios. All of the scenarios were designed to have a clear passing option but the decisions generated by players included dribbling, shooting, and holding the ball. There is evidence that viewing perspective can influence decision making (Mann et al., 2009) and it is possible that performing in an immersive environment with a first person perspective influences the options each scenario affords a player because they are able to scale their choices based on their individual action capabilities.

Although the results are encouraging, this study was not without its limitations which highlights the difficulty of working in an applied environment. The participants were the top youth players in Australia which is beneficial but also means that the sample size is going to be small. While this may cause issues with statistical power, it does need to be acknowledged that this is a limited population and a lot of insight can be gained from using these small, highly skilled groups. For example, we could expect improvements in lesser skilled groups because the landscape for improvement is much greater. Because this research was conducted in a high-performance program, there were constraints on player scheduling that needed to be worked around which is why we were not able to complete the same amount of testing and training with each group. It also limited the amount of time athletes could devote to additional training; it would be useful to determine the appropriate dose-response relationship to provide recommendations regarding the minimum amount of training needed to observe an effect. Methods of analysis need to be used (e.g., linear modeling) that take into account the likelihood that there is going to be uneven groups, and missing data due to factors like player injuries. A retention test was also not conducted under these circumstances which does not allow for statements on the long-term learning effects of the intervention and future research should include additional measures over time. From a theoretical (Craig, 2013; Hadlow et al., 2018) and design standpoint, giving the players the opportunity to interact with the immersive environment would be useful because, as the MPTF would predict, this may lead to better transfer. As previous researchers have suggested, simply providing a verbalization of the outcome and the use of a simulated movement may not have captured the full capabilities of the participants (Araújo et al., 2010; Dicks et al., 2010). Increasing the interactivity of the scenarios would require additional resources and could be beyond the capability of sporting organizations who might want to use the technology already available. Finally, the effectiveness of immersive training was not compared against any other modalities (e.g., videos presented on a monitor) so, at this stage, it is not possible to comment on the relative effectiveness of this type of training. It is possible that, relative to other training, there may be no added benefit to using immersive video training and future research should compare different approaches.

In summary, there were a number of encouraging findings in this study (e.g., improved on-court performance for trained males) along with some unexpected results (e.g., on-court improvements in the female control group). The study demonstrated that perceptual-cognitive training tools do not necessarily need to be completely representative (Davids et al., 2006) to benefit in game performance and coaches and practitioners should use a framework such as the MPTF (Hadlow et al., 2018) to understand potential trade-offs in transfer when assessing the merits of any particular training tool. Immersive training could be used for player rehabilitation and during travel to keep players cognitively engaged when they are unable to perform physically. Through the discussion a number of issues have been highlighted for future research to consider when using immersive video as a perceptual-cognitive training tool. Despite these limitations, the findings suggest that there is a potential benefit for using immersive training and it may be a practical tool that sporting organizations can implement at a relatively low cost. Given that players had positive experiences with training, enjoyed engaging with this type of presentation and there were no detrimental effects of participating in training, it is certainly worthwhile considering expanding its usage in the daily training environment.

## Implications

There were a number of practical takeaways from this research that are highlighted below:

• For development of training programs using immersive video, it is important to ensure there is enough variety in scenarios (e.g., quantity and type of decisions) to maintain player engagement. Given the accessibility and ease-of-use of technology for creating immersive content it could be quite feasible to regularly update stimuli.
• Stimuli should be created that are specific to the group engaging with the training program (e.g., female athletes should view footage of female athletes within the training footage).
• The options generated by participants in immersive environments may vary from expectations given the first-person perspective. If the goal is to target a specific aspect of decision-making skill (e.g., passing) then scenarios should be carefully designed that afford passing options over other decisions.

## Author Contributions

DP was involved in study conception and design, scenario filming, data collection and processing, analysis, and manuscript preparation. MK was involved in study conception and design, scenario creation and filming, data collection and processing, and manuscript preparation. SH was involved in scenario filming and stimulus creation, data collection and processing, and manuscript preparation.

## Conflict of Interest Statement

The authors declare that the research was conducted in the absence of any commercial or financial relationships that could be construed as a potential conflict of interest.

## Footnotes

1 http://www.fiba.basketball/3x3/rules
